# Supplementary material for: Are carbon emissions trading and green financial instruments synergistic? -Comprehensive quantitative research based on content analysis
Source: PLoS One. 2024 Mar 7;19(3):e0298601. doi: 10.1371/journal.pone.0298601 (PMC10919601; doi:10.1371/journal.pone.0298601)
Supplement: S1 Data — (ZIP) [file pone.0298601.s001.zip › Data/地级市Z_F工作报告.zip!/╣π╢1⁄2/╓╨╔╜╩╨/└┤╫╘╣1⁄2╓┌║┼í╛┬φ┐╦╩2╛▌═°í┐_2004.docx]

各位代表：

我代表市人民政府向大会作政府工作报告，请予审议，并请政协委员和其他列席人员提出意见。

过去五年工作回顾

本届政府任期五年来，在省委、省政府和市委的正确领导下，在市人大及其常委会和市政协的监督支持下，市政府坚持以邓小平理论和“三个代表”重要思想为指导，以率先基本实现社会主义现代化为总目标、总任务，按照市委“三个进一步提高、两个增强”的工作方略，大力实施“工业强市”、“科教兴市”、“依法治市”和“可持续发展”的战略，正确处理好改革、发展和稳定的关系，经济社会实现了持续快速健康发展，人民生活水平稳步提高，城市综合竞争力显著提升，全面完成和超额完成了市十一届人大一次会议确定的各项预期增长目标，社会各项建设事业取得了丰硕成果。

五年来，经济综合实力全面提升。2003年，全市国内生产总值497.1亿元，人均36278元，分别比1998年增长97.9%和86.6%，年均增长14.6%和13.3%。三大产业比例从1998年的9.47：52.29：38.24调整为2003年的5.44:62.78:31.78;出口、投资、消费分别比1998年增长202.2%、294%和77.3%。经济实力的提升主要体现在以下几个方面：一是坚定不移推进“工业立市”、“工业强市”战略，大工业管理体制基本确立并逐步完善，走新型工业化道路迈出新步伐。2003年工业总产值比1998年增长2.1倍，增幅连续三年位居珠三角首位，年均新办企业超过1000家。产业结构进一步优化，基本形成了东部高新技术、北部传统优势或特色工业、南部出口加工工业的布局。区域特色经济成为我市经济的重要特征，拥有国家高新技术产业基地、国家健康科技产业基地、国家精细化工生产基地、中国高新技术产品出口基地、中国包装印刷生产基地、中国五金制品产业基地、中国纺织产业基地市、中国灯饰之都、中国休闲服装名镇、中国红木家具生产专业镇、中国电子（中山）基地、中国电子音响产业基地、中国牛仔服装名镇等13个国家级产业生产基地。支柱产业进一步做大做强,2003年支柱产业产值（规模以上）占全市工业总产值的54.7%。高新技术产业加快发展。实行工业进园进区，开展示范园区创建活动，工业集约化水平进一步提高，全市立项建设的工业园区共40个，规划面积22万亩，已引进企业3092家。开展争创工业强镇（区）活动，镇区工业实力迅速增强，有11个镇区年工业总产值超过50亿元。推进名牌战略，开展评选中山十大名牌产品活动，2003年我市荣获“全国质量兴市先进市”称号。全市拥有中国名牌6个，驰名商标1个，国家质量免检产品17个，省名牌产品51个，著名商标37个。二是农业的国民经济基础产业地位更加牢固，效益型、城郊型农业格局基本形成，2003年全市农业总产值比1998年增长27.9%。三是第三产业有了新的发展。2003年全市第三产业增加值比1998年增长59.9%。会展经济蓬勃发展。2000年我市荣获“中国优秀旅游城市”称号。

五年来，对外开放取得新成绩。坚持用好“两种资源”，开拓“两个市场”。把握加入世贸机遇，加快与国际经济接轨，增创产业、服务和环境优势，打造全方位、宽领域、多层次的对外开放新格局。加强关贸、检贸、税贸、银贸合作，提高“大通关”水平，完善有利于扩大出口的运作机制。加大国际市场开拓力度，积极推进质量与科技兴贸战略，提高国际市场竞争力。五年累计出口202.2亿美元，年均递增24.8%。营造亲商、安商、富商的社会氛围，利用外资质量进一步提高，五年累计批准外商直接投资项目1324宗，实际利用外资29.4亿美元。目前全市有外商投资企业3500多家，世界500强企业在我市设厂20多家，长虹、TCL、小鸭、格兰仕、美的等国内大企业也在我市投资。放宽民间资本准入限制，激活民间投资，全市个体工商户和私营企业10多万户。民营企业创新能力不断增强，成为经济发展的生力军。

五年来，农业、农村经济全面发展。调整生产布局，推进农业结构调整，引进、培育和发展名、优、稀、特、新产品，建立名优产品生产基地，主要农作物良种覆盖率达到90%以上，效益稳步提高。加快农业产业化进程，大力扶持农业龙头企业，拥有省、市两级农业龙头企业共10家。推进农业标准化建设，建立了10个市级农业标准化示范基地，农业科技含量有了较大提高，农业科技贡献率达57%。完善农产品质监网络，石岐乳鸽等6个农产品荣获广东省名牌产品称号。强化农业基础设施建设。市镇两级投入2.5亿元，全面推进“一区三线”森林改造、农桥农路建设、低产鱼塘整治、“四旁绿化”和缓坡山地种果、内河整治“五项工程”。投资近2亿元建成了建国以来我市单宗投资最大的水利工程——东河水利枢纽工程，进一步改善了农村生产和生活环境。坚持“多予、少取、放活”原则，广开增收渠道，增加农民收入，减轻农民负担。取消了农村用电底度收费，实现城乡同网同价。大力推进农村税费改革，实施教育收费“一费制”，农民负担减负率达76%。采取切实措施，加强农村基层政权建设。稳步推行农村股份合作制改革；加快农村富余劳动力转移；逐步建立农村社会保障体系；推行农村财务电算化管理和会计委派制，完善村务公开等各项管理制度。开展镇区资源整合、行政村调整工作，镇区调整为24个，行政村由原来的400个调整为187个。

五年来，城市基础设施承载能力不断增强。投入36亿元开展城市建设，中心城区面积扩大到30平方公里。全市公路里程1345公里，公路密度为74.7公里／百平方公里。程控电话装机容量79.8万门，移动电话用户196.6万户，光缆长度2237皮长公里，互联网用户近30万户。年供水近7亿吨。完成了市委党校、中山大桥重建以及全民健身广场、翠山路、金字山隧道、市急救中心、岐江公园、中心组团垃圾综合处理基地卫生填埋场等重点建设工程，文化艺术中心等一批项目正在施工。做好市际、镇际交通网络规划，启动智能交通系统建设，进一步改善城市交通状况。建成了年吞吐量40万吨的神湾港码头。启动东部沿海地区开发建设研究，开展新一轮概念规划和总体规划修编工作。城镇化进程加快，城镇化水平由1998年的55%提高到60.7%。形成中心城区、中心镇、建制镇三个层次优势互补、整体协调、共同发展的格局。城镇建设“南粤杯”、“岭南杯”中期目标达标活动取得好成绩，镇区面貌明显改善。引入经营城市理念，多渠道筹集建设资金，成立了市土地储备管理中心，盘活政府性资源，提升城市自我发展能力。实施城市管理重心下移，加大旧城改造力度，完成了一批改造项目。基础设施日臻完善。

五年来，财税、金融运行秩序明显好转。财政收入稳步增长，收支保持平衡，2003年全市入库税收比1998年增长123%，年末各项存款余额和贷款余额分别比1998年初增长81.5%和68.8%。实施新的市与镇区分税分成体制，加大财政转移支付力度，调动镇区增收节支积极性。以建立公共财政框架为目标，推行预算管理制度，增强透明度、公开性和约束力；设立国库集中支付中心，强化财政支出管理；落实“收支两条线”管理制度，实施管办分离，提高资金使用效率。全面推行政府采购制度，节省资金1.1亿元。创建金融安全区工作进展顺利。通过加大力度追收资产、盘活变现、中小金融机构退出市场等多种有效手段，中小金融机构支付风险问题得到有效化解。几年来，偿还中央再贷款6.9亿元，企业集资24.5亿元，回收资产22.7亿元。境外债务清偿工作进展顺利。大力整顿和规范市场经济秩序，重点开展信用建设，联合打假，整顿旅游、文化和建筑市场，强化税收征管，整顿集贸市场、加油站等专项行动，市场经济秩序进一步好转。

五年来，各项改革进一步深化。全面启动了新一轮以公有企业产权制度改革为重点的攻坚战，按照建立现代企业制度的要求，完成了95%以上公有企业的转制工作。公有资产监督、管理、营运体系不断完善，效益提高，2003年列入考核类公有企业利润增长3.2%。政府机构改革取得预期效果，行政编制、机构、领导职数分别精简了20%、30%和11.7%，事业单位机构改革取得阶段性成果。实行公开招考录用公务员制度，推行公务员收入分配制度改革。开展了三轮政府审批制度改革，共减少审批事项1000多项，减幅75.1%，下放审批权限，减少审批环节，推行“窗口式”、“一站式”办公，行政成本进一步降低，效率进一步提高。

五年来，人民生活水平和生活质量明显提高。2003年城镇居民年人均可支配收入、农民年人均纯收入分别较1998年增长43.8%和51%。初步建立了以社会养老、失业、医疗、工伤及生育保险等五大险种配套，覆盖全社会的社会保障体系。各类险种参保270多万人次，低保对象基本实现了“应保尽保”。初步建立了与低保相配套的医疗救助、住房解困、教育救助、法律援助等城乡社会救助体系。人居环境和市民生活不断改善。建成解困房近4万平方米，基本解决了城区双困户住房问题。旧城改造总面积9万多平方米，城区居民人均居住面积达30平方米，农村居民人均居住面积达34.2平方米。新建住宅小区197个，绿化覆盖率达到38.3%，城区人均绿地面积10.78平方米。加大环境治理力度，切实做好创建国家级生态示范区，扎实推进工业污染源达标排放和水环境综合整治以及水、空气、噪音等综合整治，城市综合定量考核总分连续5年位居全省前5名，可持续发展能力进一步增强。全面整治农地违章建筑和沙、泥、石场，土地资源保护和集约开发得到重视和加强。

五年来，社会各项事业发展跃上新台阶。教育方面，基本普及了高中教育和学前三年教育，初中升高中升学率91.8%，高等学校毛入学率29%，基础教育水平稳步提高。成都电子科技大学、广东药学院等高校在我市办校，高等教育有了新的发展。启动教育人事制度改革，调整学校布局，推进名师、名校、名校长三大工程，教育综合实力明显提高。科技和人才方面，实施“人才柔性流动”战略，引进各类人才12.8万人。深化科技管理体制改革，科技创新和技术进步能力明显提高，获得省以上科技成果62项，承担国家“863计划”5项，高新技术产品产值占全市工业总产值比1998年提高了11.5个百分点。在2002年全省年度科技创新能力评估中，我市位居第5位。各级高度重视科技投入，市本级科技三项经费累计2.8亿元，占财政支出的2.2%，技改贷款贴息累计9600万元，拉动投资77.3亿元。加强产学研合作，全市各类研发机构90多个。中山民营科技园被评为“全国十佳民营科技园”，小榄、古镇、沙溪、大涌、南头、黄圃、民众、东凤8个镇成为“广东省科技创新专业镇”。文化方面，发挥“伟人故里”优势，提高城市文化品位，重建孙中山故居纪念馆，建成中山城、全民健身广场等一批文化体育设施。全民健身运动蓬勃开展，竞技体育取得优异成绩，获得4个世界冠军、8个亚洲冠军、30多个全国冠军，蝉联省第十届、第十一届运动会团体总分和金牌总数第3名的好成绩。卫生方面，开展卫生事业改革，整合医疗资源，公共卫生体系逐步完善。计划生育方面，完成了省下达的人口控制指标，计生服务质量上了新水平。精神文明各项创建活动卓有成效，获得了“广东省文明城市”和“全国创建文明城市工作先进市”称号。加强民兵预备役建设和国防教育，双拥工作取得优异成绩，荣获“全国双拥模范城”荣誉称号。

五年来，民主法制和政府自身建设取得新成效。积极应对加入世贸需要，清理规范性文件1100多件。坚持重大事项向市人大及其常委会报告制度，坚持市政府领导与人大、政协领导座谈及重大决策听证等制度。加强机关作风建设，提高公务员素质，行政效率不断提高。开通了中山市党政信息平台，坚持市长接待日制度，开通市长专邮和电子邮箱，在《中山日报》开办政务专版，实行政府新闻发布制度，政务、村务、厂务公开不断深入，公开水平进一步提高。大力开展社会治安综合治理，落实各级信访责任，切实抓好安全生产，社会大局保持稳定。

民政、监察、外事、侨务、台务、审计、统计、老干、信访、广播电视、气象、国防建设、档案、地方志、妇女儿童、残疾人、民族宗教、新闻出版、人防、老区建设等各项工作取得了新的成绩。

2003年是全面建设小康社会的第一年，也是我市经受严峻考验，战胜困难，取得显著成绩的一年。全市上下认真贯彻落实市委十届六次全会精神，围绕市十一届人大六次会议确定的目标任务，抓住机遇，奋力拼搏，经济和社会事业持续快速健康发展。一是取得了“抗非典”和“谋发展”的双胜利。面对突如其来的非典疫情，我们把保护人民群众的身体健康和生命安全放在第一位，采取了一系列行之有效的措施，坚持一手抓经济，一手抓非典防治，打赢了一场没有硝烟的战争，经济总量和质量同步提升。全市国内生产总值增长17.8%，实现工业总产值1767.5亿元（不变价），增长31.3%；农业总产值34.1亿元（不变价），增长4.1%；外贸出口82.5亿美元，增长44.2%；实际利用外资9.5亿美元，增长23.8%；一般预算财政收入36.6亿元，增长19.9%；全社会固定资产投资261.1亿元，增长19.2%；社会消费品零售总额151.7亿元，增长12.9%。年末各项存款余额和贷款余额分别比年初增长19.4%和12.1%。工业增长质量和效益同步提高，一批新项目相继落户，园区建设水平不断提高；农业夺得丰收，以农民增收为核心的农村工作取得新成效。二是各项改革取得新进展。按照“有所为，有所不为”，“有进有退”的原则，大力推进公有企业和生产经营型事业单位改革，审批制度、事业单位分配制度改革取得成效。建立公共财政框架工作进一步推进。三是城乡建设取得新成果，一批基础设施项目相继建成，城市承载力进一步增强。四是人民生活水平稳步提高，社会事业取得新进展。城镇居民年人均可支配收入14906元，增长4.9%；农民年人均纯收入6520元，增长10.8%。各级财政增加资金投入，进一步提高低保对象的保障标准，社保体系逐步完善。高度重视城乡就业问题，市级财政安排1600万元，实行城乡就业培训补贴等多项扶持性政策，城乡就业新增5.3万人，城镇登记失业率控制在2.5%以内。关注困难群体和残疾人事业发展。全面启动“安全五项工程”建设。科技、教育、文化、卫生、体育等各项事业进一步发展，精神文明建设迈上新台阶。我市获得“全国科技进步先进市”称号并通过“全国园林城市”复核。全年常住人口出生率为10.4‰，计划生育率93.5%。

各位代表，过去五年，是我市经济取得快速健康发展，人民群众得到实惠较多的五年；是城市综合竞争力日益增强，现代化水平不断提高的五年；是各项社会事业全面发展，社会主义政治文明和精神文明不断取得新进步的五年。在五年的工作中，我们深深地体会到：

——必须坚持党的领导，坚持以邓小平理论和“三个代表”重要思想为指导，解放思想，实事求是，与时俱进，开拓创新，坚定不移地把发展作为第一要务，聚精会神搞建设，一心一意谋发展，正确处理改革、发展、稳定的关系，依靠发展解决前进道路上的困难和问题。

——必须坚持依法行政，执政为民，把改善民生，实现人的全面发展作为政府工作的出发点和落脚点，切实转变政府的工作作风、工作方式和工作方法，高度重视和解决好人民群众普遍关心的热点和难点问题，为人民办好事、办实事。

——必须坚持以改革为动力，全面深化公有产权制度、政府机构、社会保障体系等各项改革，加快建立和逐步完善社会主义市场经济体制，增强经济社会发展的源动力，不断实现新的跨越和新的发展。

——必须坚持走新型工业化道路，坚定不移推进工业强市战略，推动经济结构和产业结构的优化升级，促进三大产业协调发展，努力做大经济总量。坚持全面开拓国际国内两个市场，努力扩大有效需求，拓宽经济发展空间。

——必须坚持走可持续发展道路，正确处理好经济建设与人口、资源、环境保护的关系，实现经济社会的协调发展。

——必须坚持“两手抓”，把政治文明、精神文明建设摆在更加突出的位置，实现三个文明同步发展和社会全面进步。

各位代表，我市五年来取得的成绩，是省委、省政府和市委正确领导的结果，是市人大及其常委会依法监督，市政协以及各民主党派民主监督和支持的结果，是全市人民群众努力奋斗的结果。我谨代表第十一届市政府向全市广大工人、农民、知识分子、干部职工、驻我市人民解放军指战员、武警官兵、人民警察和各界人士表示崇高的敬意，向长期关心支持中山建设的港澳台同胞、海外侨胞和国内外友人表示衷心的感谢！

我们也清醒地看到，我市经济社会发展还存在一些突出问题。主要是：经济总量不大，区域发展不平衡；基础设施建设与经济社会发展的要求不相适应；产业优势不够明显，产业结构调整力度还需加大;市场经济体制还需进一步完善；农村基层建设仍需进一步加强；影响社会稳定的因素仍然存在；政府职能的转变和审批制度改革有待进一步推进等。这些问题必须引起高度重视，采取有力措施加以解决。

今后五年的展望和2004年工作建议

今后五年，是全面建设小康社会、率先基本实现社会主义现代化和建设全省经济社会协调发展示范市的关键时期。必须深刻认识经济社会发展所面临的机遇和挑战。当前，世界经济一体化进程不断加快，国际经济技术交流与合作更加广泛。我国经济正处于经济周期的上升阶段，经济自主增长能力不断增强。内地与港澳建立更紧密经贸关系为广东经济发展带来了新的机遇。同时，必须看到，我市原有的一些发展优势正在减弱，地区之间的竞争日趋激烈，形势逼人，不进则退。我们必须树立强烈的忧患意识，以高度的政治责任感和历史使命感，树立科学的发展观，更加注重经济运行的质量和效益，更加注重城乡和镇区之间的平衡发展，更加注重三大产业的协调发展，更加注重内外源经济的同步发展，更加注重经济社会与生态、环境的可持续发展，更加注重人民生活水平的提高，继续增创产业、体制、开放、科技和环境优势，努力推动我市经济社会实现新的跨越。

今后五年政府工作的总体要求是：高举邓小平理论和“三个代表”重要思想伟大旗帜，全面贯彻党的十六大、十六届三中全会、省委九届四次全会和市委十届七次全会精神，按照“三个进一步提高、两个增强”的工作方略，树立科学的发展观，把工作重点转到调整经济结构、转变增长方式、提高增长质量和效益上来，加快从增量型经济向效益型经济的转变，增创城市经济新优势，实现加快发展，率先发展，全面、协调、可持续发展。加快体制、科技创新，全面提升城市综合实力。坚持工业强市、科教兴市、依法治市和可持续发展战略，坚持以人为本，提高市民素质，提高群众生活质量，促进社会主义物质文明、政治文明和精神文明同步发展。全面建设小康社会，加快率先基本实现社会主义现代化，努力把我市建设成为广东省经济社会协调发展示范市。

未来五年政府工作的奋斗目标和主要任务是：

——城市经济综合实力有较大提升，产业强市初具雏形。经济总量、质量同步提高，区域集群产业优势更加凸现，三大产业协调发展。工业化、信息化、城镇化实现良性互动。主要经济指标达到或接近现代化水平。

——城市基础设施和生态环境体系逐步完善，区域性中心城市的地位进一步加强。城市生态质量指标达到国内先进水平。

——全方位对外开放格局进一步扩大。更加主动承接世界产业结构转移，积极参与泛珠三角经济技术合作与竞争，对外贸易竞争力明显提升。利用外资质量稳步提高，服务业领域进一步扩大开放。

——社会主义市场经济体制更加完善。公有资本、集体资本和非公有资本参股的混合所有制经济更显活力；归属清晰、权责明确、保护严格、流转顺畅的现代产权制度基本完善；统一开放，竞争有序的现代市场体系逐步形成。

——人民群众生活水平显著提高。建立起覆盖全社会的社会保障和就业体系，城镇登记失业率控制在3%以内。市民的物质和精神需求得到更大的满足。城镇居民年人均可支配收入和农民年人均纯收入持续增长。

——物质文明、政治文明和精神文明协调发展。科技、教育、文化、卫生、体育等各项事业与经济发展同步推进；社会公德、职业道德和家庭美德教育深入人心；学习型社会、健康型城市特色更加鲜明，城市文化品位进一步提高；市民文明素质全面提高。

2004年是全面贯彻落实党的十六届三中全会精神，实现“十五”计划各项预期目标的重要一年，做好今年工作意义重大。2004年我市经济社会发展的主要预期目标是：全市生产总值增长13%左右，工业增加值增长15%，社会消费品零售总额增长12%，固定资产投资增长11%，外贸出口增长10%，实际利用外资增长8%，居民消费价格总指数上升1%左右，城镇居民年人均可支配收入增长8%，农民年人均纯收入增长7%，城镇登记失业率控制在3%以内，人口自然增长率控制在5.7‰以下。

为实现上述目标，必须着力抓好以下工作：

一、 积极参与区域分工，加快实施产业强市战略

推进产业结构优化升级，增创产业集群优势。按照走新型工业化道路的总体要求，抓住全球产业转移的机遇，积极参与大珠三角产业分工，大力推进工业强市、产业强市战略，努力建设各大产业分工协调，特色经济充满活力，支柱产业支撑作用更加突出，产业布局协调集聚的产业强市。全面提高装备工业发展水平，提升工业的核心竞争力和整体实力。加大产业结构调整力度，加快从轻加工工业向高加工工业阶段跨越。大力发展高新技术产业，办好中国(电子)中山基地和国家健康科技产业基地，强化行业整合，努力把我市建设成为国内重要的电子信息制造业基地和珠三角IT产业带中的重要一环，高新技术产品产值占工业产值的比重达到22%以上。

做大做强支柱产业。依托我市产业基础和资源优势，扶持电子信息、电器机械、化工制品、纺织服装和金属制品五大支柱产业发展，提高产业竞争力和发展后劲。发展医药、包装印刷、汽配等行业，提高国家级精细化工生产基地、健康科技产业基地和包装印刷基地的竞争力。积极培育和引进软件、新材料、新能源、环保和光机电一体化等高成长度产业，培育新的产业增长点。按照“小商品、大市场，小产品、大产业”的思路，做大做强特色产业，增创产业集群优势。完善政策引导和社会化服务体系，建设好五金、音响、纺织、服装、家具、食品、家电等专业化生产基地，形成一批特色突出、辐射力强、技术先进、产业配套、经济效益好的特色产业集群，进一步整合产业资源，延伸产业链条，增强产业竞争力。

抓好工业园区建设，推进工业集约化发展。编制2004——2010年工业园区发展总体规划，明确定位，优化布局。制定园区投资密度和产出效益标准，提高园区建设水平。多渠道解决园区建设资金，加快园区集约发展步伐。

实施龙头带动和名牌战略，增强工业核心竞争力。制定和完善装备制造业发展规划，坚持引进、消化吸收与自主创新相结合，政策支持与市场竞争相结合，资本投入与人才培养相结合，发展一批装备制造业重点企业。打破地区、行业、所有制界限，通过企业购并、资本技术联合和引进等手段，培育技术先进、规模较大、辐射带动力强的大型企业集团。继续推进名牌战略的实施，加大政策引导，切实加强对名优品牌的法律保护，争创一批省级以上名牌产品、著名或驰名商标、国家质量免检产品，提升中山产品竞争力。

二、统筹城乡发展，提高三农工作整体水平

大力发展效益农业，努力实现农民增收。深入贯彻落实中央1号文件精神，把解决“三农”问题，发展农村经济放在重要位置，发挥城市对农村的带动作用，促进城市和农村相互促进、协调发展，确保实现农业生产总值增长3.5%。大力调整农业产业结构，发展特色农业、品牌农业、创汇农业和绿色农业，走大规模、精加工、大市场的路子，大力发展效益农业。推进农业产业化经营，提高标准化、集约化水平，提高农产品科技含量和附加值。培育一批龙头企业，鼓励和支持农业龙头企业申请自营进出口经营权。发展农民专业合作经济组织，提高农业的组织化程度和产出效益。加大从源头上减轻农民负担的力度，深化粮食流通体制改革，继续落实农村税费改革政策，抓好农民负担监督管理的“七项重点工作”。推进涉农价格公示和教育收费“一费制”工作。

加强农业基础设施建设，推进农业现代化进程。组织实施好农村“五项工程”（一区三线森林改造、“四旁”绿化和缓坡山地种果、农桥农路建设、鱼塘整治、内河整治），掀起第二轮建设高潮，加强后续管理，巩固成效。抓好重点险闸、险堤、险段、险库改造加固工程，提高防灾减灾能力。切实抓好省、市农业现代化示范区建设。

积极发展和壮大农村集体经济。完善市与镇区分税分成管理体制，继续加快以体制和专项补助为重点的财政转移支付体系建设，促进镇区平衡和协调发展。积极推进农村股份合作制，支持和帮助农村集体经济组织建立现代企业制度。大力发展村级集体经济，一村一策，因地制宜，帮扶经济薄弱村增加经济收入。

加强农村基层建设，确保农村大局稳定。健全基层自治组织，完善村民自治和居民自治。加强村级民主决策和民主监督工作，完善村务监督制度，加强村级集体经济审计与监督，扩大村务公开，深入开展“三级联创”活动。切实加强教育培训和作风建设，增强农村基层干部法制意识，规范村级干部收入管理，提高依法行政能力。提高村集体资产管理水平，全面推行村帐镇管、会计电算化和会计选聘制度。

三、把握粤港澳合作新机遇，促进内外源经济快速协调发展

积极参与国际竞争与合作，全面提升对外开放水平。承接新一轮国际资本和产业转移，努力营造低成本、高效率、文明法治的投资环境。高度重视投资环境整体推介，创新招商引资方式，积极探索市场化、产业化招商模式。坚持规模与质量并重，重点引进关联度大、聚集度高、带动作用强的龙头型、基地型、技术密集型项目，引进跨国公司的生产中心、采购中心、维修中心、研发中心和营销中心。鼓励现有外资企业增资扩股。继续推进科技兴贸、以质取胜和市场多元化战略的实施，增强出口产品的科技含量和市场竞争能力，努力扩大出口。积极推行质量管理体系认证和出口产品国际标准认证，加快与国际环境标准接轨的步伐。抓好大通关建设，继续提高通关效率。

主动适应内地与港澳更紧密经贸合作，提升第三产业发展水平。按照扩大总量、优化结构、拓宽领域、提高水平的要求，加快第三产业对外开放步伐，构筑布局合理、科技含量高、辐射能力强的第三产业自我发展体系。加快服务业市场化、社会化步伐。大力发展现代服务业，加强与港澳地区服务业的合作，加大引资力度，培育金融、证券、保险、中介和专业服务业。加快物流业发展。完善以公路骨架网为主的综合运输网络，挖掘港口吞吐潜力，进一步提高货运能力。加快现代物流基础设施建设。

大力改造和提升传统服务业。放宽市场准入，鼓励发展连锁经营、多式联运、网上销售等组织形式和服务方式，提高服务质量和经营效益。积极引导和培育新的消费形式。发展以经济适用住房为重点的房地产业，推广和规范物业管理。大力发展旅游产业，鼓励和支持旅游设施建设，开发和保护旅游资源，丰富旅游产品。加快发展社区服务业，提供便民利民服务。引导文化娱乐、教育培训、体育健身、卫生保健等产业发展，满足服务性消费需求，提高传统服务业的技术含量和对经济增长的贡献。

扩大投资需求，增强内源型经济活力。挖掘民营资本的巨大潜能，培育充满活力的市场主体。鼓励民营资本投资基础设施、公用事业及其它行业和领域，按照“四个不限”的原则，放宽民营企业登记注册条件，简化程序、下放权限，开辟民营资本投资发展的“快速通道”，确保民营企业在投融资、税收、土地使用和对外贸易等方面享受其它类型企业的同等待遇。加强知识产权保护、市场监管和中介服务，推进科技型、外向型、农产品加工型、就业型民营企业的发展。加快中小企业服务体系建设，发展行业协会和中介机构，构建多形式、多层次、全方位的中小企业服务网络。

四、加快功能型基础设施建设，全面提升城市承载能力

坚持高起点规划和建设，提升城市综合竞争力。抓住珠三角新一轮规划调整的机遇，按照互补、错位、共享，合作、共建、共赢的原则，加强总体和专项规划，按照完善功能，拉大城市框架的思路，加快全市总体战略研究与概念规划编制工作，为新一轮经济社会发展奠定基础。按照建设珠江口西岸区域性中心城市，重要的现代制造业基地，宜于居住和创业发展，经济和社会协调发展的示范市的总体目标，全面提升城市规划、建设水平。积极推进东部沿海地区开发步伐，加快建设东部经济走廊，打造新的经济和科技辐射源，培育新的经济增长极。完善城市规划体系建设，增强规划的权威性、严肃性，把规划管理纳入法治化轨道。

建设一批城市功能型基础设施。按照“适度超前、突出重点、城乡一体、配套完善”的要求，加快构筑系统化、网络化、现代化的基础设施体系。科学规划建设一批功能型项目，带动产业布局的战略性调整和社会事业的进一步发展。按照“内通外拓”的思路，加快与周边城市交通网络的对接，配合省做好轻轨和“两纵两横一环”高速公路网建设准备工作。启动“五纵五横”路网规划，加快镇际公路建设步伐。重点推进古神公路、南沙公路、沙水路、福源路的建设以及番中公路、岐江公路改建工程，力争早日实现“3015”目标（30分钟从中心城区到达镇区，镇区15分钟内可上高速公路）。整治城市出入口。推进城区污水收集管网等项目建设，启动南部、北部组团垃圾处理基地项目，加快推进香山550千伏输变电工程及逸仙、旗乐和同益三个220千伏输变电站建设，确保年内投产，新建坦洲、小榄西、新隆及黄圃四个220千伏输变电工程，完成蒲鱼洋10万吨水厂迁（扩）建工程。

逐步完善中心城区功能，增强辐射带动能力。重点推进文化艺术中心、会展中心、长堤路改造、污水处理厂、垃圾组团处理、危房改造、疾控中心、第二人民医院等工程。建设一批休闲广场和生态公园，推进“城中村”改造。完善智能交通系统，改造博爱路、起湾道、孙文中路、民权路、民生路等城区道路，完善交通基础设施，按照公交优先的原则，解决交通拥挤问题。

推进组团发展，提高城镇化水平。以规划为导向，推进中部、南部、西北部和东部等四大组团发展，通过对产业、资源的有效整合，明确发展方向，促进组团之间、组团内各镇之间实现优势互补、资源共享、产业联动和共同发展。进一步加大城镇公用事业及各项基础设施建设和管理的力度，全面提升城镇建设的总体水平。强化镇、村规划的“三区”、“六线”管理，增强中心镇联结城乡的纽带桥梁功能，建设要素聚集明显、发展特色鲜明的现代城镇。大力推进各种跨镇、跨村、相邻地域的市场设施建设、污染治理、环卫管理、绿化维护的共建、共享试点工作。推进户籍管理制度改革，引导农村富余劳动力实现产业转移、地域转移和劳务输出，增强中心镇对人流、物流、资金流、技术流等各类生产要素的集聚和承载功能，以中心镇、中心村建设推动城镇化步伐。

创新城市管理和投资体制。深化城市管理综合体制改革。坚持城市管理与行政执法的有机结合，推进“重心下移”。树立经营城市的理念，通过重组、积聚和营运等手段，降低准入门槛，引入竞争机制，多渠道筹集建设资金，推进市政公用事业产业化、市场化进程。调整和完善城市基础设施政策性收费和价格体系。加强土地储备中心建设，完善土地储备制度。

五、深化改革和制度创新并重，全面增创体制新优势

完善公有资产营运管理体系。加快公有企业实行以股份制为主要形式的改造，推进股权多元化，大力发展混合所有制经济，使股份制成为公有制的主要实现形式。依法保护各类产权，健全产权交易规则和监管制度，推动产权有序流转，保障所有市场主体的平等地位和发展权利；完善公有资产管理体制，规范重组后的公有资产营运体制，盘活沉淀资产，加快债务重组以及资产的流动和变现，确保公有资产安全增值，提高资产营运效率。落实公有资产管理机构作为出资人的职责，逐步建立公有资产经营预算制度业绩考核体系，实施对公有资产的有效监管。培育和发展资本、产权、劳动力、土地等要素市场，充分发挥市场对资源配置的基础性作用。按照完善市场经济体制的要求，加快推进各项事业改革，做好新一轮政府机构改革工作。

完善财税管理体制，加快建立公共财政框架。健全财政管理制度，加强对财政拨款的绩效评估，调整优化支出结构，保证重点支出需要。全面实行部门预算改革，开源节流，实行国库集中支付，积极组织非税收性财政收入。加大管理和稽查力度，整顿和规范税收秩序。规范政府采购行为，扩大政府采购覆盖面，提高财政资金使用效益。建立市场引导投资，企业自主决策，融资方式多样，中介服务规范的新型融资管理体制。

优化市场经济秩序，建设“信用中山”。大力整顿和规范市场经济秩序，严厉打击制假售假、商业欺诈等违法行为，深入开展反走私斗争，严厉打击非法和变相传销活动，规范特殊行业管理。加大力度建设金融安全区。加快社会信用体系建设，建设以道德为支撑、产权为基础、法律为保障的社会信用制度。探索企业信用公示制度，建立奖惩机制，建设诚信社会。

六、推进科教兴市战略，增强经济社会发展后劲

完善创新体系，提升科技竞争力。完善以企业为主体的技术创新体系，打造高新技术产业集聚和提升平台，抓好“一区一园三基地”建设，年内力争省级高新技术企业达到100家，省级民营科技企业达到60家。引导专业镇增强创新能力，扶持镇区争创省级试点镇。强化工程技术研究开发，积极组建一批广东省工程技术研究开发中心。抓好100项技术创新和改造工程。建立多元化科技投入机制，激发各类科技投资主体的积极性，培育和引进风险投资机构。发挥财政资金的放大作用，确保科技三项经费增长。落实国家、省、市各级有关技改和软件发展的政策，支持企业技改和软件开发。培育和发展中介组织，建立健全与科技创新活动相适应的市场机制。

增加人力资源储备。高度重视人才资源在经济社会发展中的基础性、战略性、决定性作用，营造尊重知识、尊重人才、尊重创造，促进人才成长的良好环境。健全各类人才选拔、任用、考核、评价、激励、监督机制。坚持“以人为本”，按照各类人才成长规律和不同特点去识别和使用人才，坚持任人唯贤、唯才是举，使各类人才创业有机会、干事有舞台、发展有空间。建立规范高效的人才服务体系，加强人才市场的培育和管理，建立人才需求信息预报机制，畅通人才流动渠道，完善人事代理、人才租赁、人事仲裁等制度，建立人才评定机制，实行人才科技成果奖励制度。全市人才总量增加到26.9万人。

推进教育改革，加快建设教育强市。落实分级办学、分级管理体制，加大投入，确保教育经费增幅与财政支出增幅基本同步，协助镇区筹集资金办教育，提高教育发展水平。积极发展民办教育，增强教育发展活力。深化中小学人事制度改革，推进“三名”工程，开展校长职级制改革，加快形成充满生机和活力的用人机制。坚持调整学校布局与积极推进等级学校、示范性学校建设相结合，加快建设规范化学校步伐。启动振兴初中行动计划，扩大普通高中和职业中学优质教育资源，力争初中升学率达92%以上，高等教育毛入学率达32%。协助电子科技大学、广东药学院等高等院校办学，大力发展幼儿教育、中高等职业技术教育，完善国民教育体系和终身教育体系，积极建设学习型社会。

发展先进文化，建设文化强市。树立“文化资本”观念，提高城市文化品位，增强城市文化竞争力。创新投资机制，完善文化产业政策，优化整合文化资源，引进和培养文化人才，繁荣新闻、出版、图书、影视事业，培育文化产业。切实保护和深入挖掘历史文化资源和优秀民间艺术，加强文物、档案资源的保护和开发利用。完善城乡文化设施建设，完成商业博物馆建设。加强文化市场管理。推动对外文化交流。

七、优化人居和生态环境，进一步提高人民生活水平

大力推进城市生态环境建设。以全面、协调和可持续的科学发展观统筹经济与社会发展，努力建设一流的人居和生态环境、人与自然和谐共存的生态文明。加快规划和建设中嘉污水处理厂二期、珍家山污水处理厂一期和镇区的生活污水处理厂。城区全面推广垃圾分类收集，并积极向镇区推广，加快建设全市医疗卫生废物集中处置基地。加强“城中村”环卫管理。深入开展园林绿化工作，高标准建设环城道路的防护绿带，重视城市绿化植物的选择，重视城市绿化生态性与城市景观的有机结合，逐步形成多树种、多层次、多功能，点线面相结合的森林生态体系。严格控制水、噪音、大气污染，抓好污染源的达标排放和全程监控管理。推进水环境综合整治工作，加大对内河、工业污染源等的整治力度。落实国家土地资源保护政策，整治采石（泥）场、沙场、陶土场、实心砖厂，实施复绿、复耕工程。建立和完善环保产业的政策支持体系，推广清洁生产，大力发展生态产业和环保健康产业。

建立健全疾病预防控制体系。加快推进一套反应灵敏、行动迅速的突发公共卫生事件应急预案，一个完善的公共卫生信息系统，一套健全的公共卫生服务架构，一支专业素质较强的公共卫生服务队伍，一个确有保障的公共卫生投入机制的“五个一”工程，加强公共卫生基础设施建设，完善运行机制，提高对突发公共卫生事件的应变能力。建设城市卫生管理信息平台和疫情信息系统，全面提高防控能力。提高社区卫生服务水平和质量，建设以防为主，重心在社区的服务体系。深入开展爱国卫生运动，突出做好非典等传染病的防治工作，严防禽流感感染到人。扩大农村合作医疗覆盖面，确保覆盖面达到90%以上，整合镇区卫生资源，强化镇区医院职能，推进镇、村医疗卫生一体化管理，加强农村社区卫生服务网络建设，提高保障能力。加大对农村卫生工作的投入，鼓励社会力量参与农村卫生服务建设，活跃医疗市场。支持和发展中医中药事业，加强专科建设，提高医疗卫生质量和水平。

推进安全五项工程。全面组织实施城乡水利防灾减灾工程，扎实开展水利建设“三大会战”。完成一批危桥（涵）改造。加快城区公房危房改造，完成4.5万平方米旧房拆迁工作。抓好疾病防控体系的重点工程。加快绿色食品基地建设，加强农产品安全监测，确保食品安全。

推进城乡就业和社会保障工作。把扩大就业摆在经济社会发展更加突出的位置，建立统一的城乡劳动力市场，完善城乡劳动者平等就业的制度。建立和完善职业培训网络，采取多种方式，加强职业技能、法律等知识培训。提供就业信息、政策咨询、就业指导、职业介绍等服务，计划新增就业岗位5万个。深化社会保障制度改革，继续做好“两个确保”工作，加大社保扩面力度，确保参保总数达到300万人次，其中，养老保险参保达到72万人。完善劳动争议调解网络，加大监察力度，保障劳动者合法权益。完善农村社会保障体系，确保低保对象实现动态管理下的应保尽保。逐步提高低保对象、下岗职工的保障水平。完善对低收入者的救助制度，提高社会救助水平。开展面向困难群众的救济、援助活动，为困难群众提供法律援助。建立困难学生财政资助制度。大力发展残疾人事业，力争两年内残疾人工作进入全省先进行列。加快新一轮住房解困工作。

八、坚持协调发展，促进各项社会事业再上新台阶

大力提高市民素质。深入贯彻公民道德建设实施纲要，广泛开展群众性精神文明创建活动，加强社会公德、职业道德和家庭美德教育，努力提高人民群众道德观念、法制意识和文明素质。进一步加大文明社区、文明村镇、文明窗口创建工作的力度，继续做好“法律、科技、文化、卫生、体育”进社区的活动。积极引导市民摒弃各种不文明行为，推动良好社会风气的形成和良好道德习惯的养成，努力实现人的全面发展。

确保社会稳定。完善基层信访网络，落实“关口前移”，落实重大信访领导包案“五个一”责任制，加大对苗头性、倾向性问题的调处力度，认真兑现政策，从源头上解决矛盾纠纷。高度重视社会治安工作，完善群防群治网络，保持对各种危害社会治安的刑事犯罪和黑恶势力的“严打”态势，查禁“黄赌毒”，全面推进禁毒“03工程”；加强对出租屋、娱乐场所的管理。构建现代警务机制，加强公安、司法队伍建设。切实抓好安全生产工作，严查隐患，堵塞漏洞，确保安全生产形势平稳，维护社会稳定。

加快社会各项事业发展。加强人口、计划生育工作，推进创建计划生育优质服务先进镇（区）活动，确保完成省下达的人口计划。发展体育产业，构建更加完善的体育设施服务体系，提高全民健身运动和竞技体育水平。加快社会福利事业发展，加强各项社会行政事务管理。推进社区建设，打造管理完善、设施完备、健康安全的社区环境。进一步加强国防教育，抓好民兵预备役工作。提高双拥工作水平。大力发展妇女、儿童、老龄人、残疾人等各项社会事业。

九、努力建设服务型、效率型、责任型、法治型政府

以政府服务效率提速为重点，进一步优化政务环境。把政府自身建设摆在更加重要的位置。切实加强机关作风建设，树立以人为本的政府管理理念，努力建设廉洁、高效、务实、勤政的“服务型、效率型、责任型、法治型”政府，加快从行政管理向公共服务转变，提高公共产品和公共服务质量。按照省委、省政府把今年确定为“抓落实，促发展年”的各项要求，切实抓好政府行政效率提速。加快电子政务建设，进一步完善党政信息网络平台，加大电子政务数据资源整合和共享的力度，争取90%的政府部门实现“网上办公”。推进新一轮审批制度改革，缩短审批时限，降低行政成本。完善新闻发布制度，最大限度实行政务公开，提高决策透明度。

坚持依法行政。由注重依靠行政手段管理向注重运用法律手段管理转变，全面落实行政执法过错追究责任制度，逐步推行行政执法评议考核制度，建设“诚信政府”，提高政府公信力。尊重和维护市民对政府决策的“知情权”，扩大重大决策听证范围。坚持重大事项向人大及其常委会报告制度，加强与人民政协的联系，完善市人大代表建议在线办理系统，认真办理各项议案、建议和提案。充分发挥民主党派、工商联、无党派人士参政议政和实施民主监督的作用，拓宽人民群众参与社会事务管理，监督政府工作的渠道。进一步加大政府督办工作力度，确保政令畅通。做好实施《行政许可法》各项准备工作。

加强公务员队伍建设。在公务员队伍中广泛开展“两个务必”教育，坚持执政为民，干净干事，做到“权为民所用、情为民所系、利为民所谋”，努力打造一支想干事、会干事、干成事的公务员队伍。继续开展机关作风评议以及行风评议等活动。加强政风、廉政、职业道德建设，从源头上预防和治理腐败，形成更加完善和有效的法律、舆论、群众和层级监督机制。

各位代表！时代赋予我们神圣使命，重担在肩，任重道远。让我们在省委、省政府和市委的领导下，高举邓小平理论和“三个代表”重要思想伟大旗帜，按照市委十届七次全会的部署，与时俱进，开拓创新，以奋发有为的精神状态、求真务实的工作作风，为全面建设小康社会、率先基本实现社会主义现代化，为把我市建设成为全省经济社会协调发展示范市而努力奋斗！
